# REVIEW OF MEDICATION INCIDENTS IN MENTAL HEALTH SERVICE

**DOI:** 10.1192/j.eurpsy.2024.1482

**Published:** 2024-08-27

**Authors:** T. C. Gomes

**Affiliations:** Psychiatry, Tallaght university hospital, Dublin, Ireland

## Abstract

**Introduction:**

In this review, medication incidents accross different mental health care facilities was reviewed and nuances, challenges, and advancements in the administration and management of psychiatric medications was noted. Through gaining a better understanding of the complexities surrounding these incidents, valuable information can be gathered that will enhance patient safety, improving healthcare practices, and fostering a deeper understanding of the critical intersection between mental health care and medication management.

**Objectives:**

To identify the most frequent types of medication errors or patterns of medication errors in a mental health service accross different settings including inpatient, outpatient, liaison and long term residential unit

**Methods:**

This is a multicentre project as it covers medication incidents in mental health care in a regianal area in Ireland. It includes an acute psychiatric Unit, the General Hospital and patients admitted in medical and surgical wards and as well long term residential care. Using the National Incident Management System we collected National Incident Report Forms (NIRF) relating mental health care provided and medication prescribed within a region in Ireland. From these we selected the ones were medication hazard was noted. Data collection happened between July 2020 and July 2021. A statuystical analysis was then performed to identify any patterns to medication errors.

**Results:**

A total of 22 incidents were included. On review of these, it was noted, among other findings, that here was a significant increase in the frequency of medication errors during the month of December. It was also noted errors ranged from medication being given to the wrong patient, medication being given twice and medication being missed.

**Conclusions:**

Minimising medication errors requires a comprehensive, multidisciplinary approach that involves healthcare providers, patients, and healthcare systems. Healthcare organizations should foster a culture of safety where medication errors are seen as preventable and where providers are encouraged to report errors without fear of retaliation.

**Disclosure of Interest:**

None Declared

